# Substance Use Among Young Mothers: An Analysis of Facebook Posts

**DOI:** 10.2196/10261

**Published:** 2018-12-04

**Authors:** Daniel Oram, Golfo Tzilos Wernette, Lauren P Nichols, VG Vinod Vydiswaran, Xinyan Zhao, Tammy Chang

**Affiliations:** 1 Department of Family Medicine School of Medicine University of Michigan Ann Arbor, MI United States; 2 Institute for Healthcare Policy and Innovation University of Michigan Ann Arbor, MI United States; 3 Department of Learning Health Sciences University of Michigan Ann Arbor, MI United States; 4 School of Information University of Michigan Ann Arbor, MI United States

**Keywords:** adolescents, Facebook, mobile phone, pregnant, substance use, social media

## Abstract

**Background:**

Substance use among young pregnant women is a common and significant public health concern associated with a number of adverse outcomes for both mothers and infants. Social media posts by young women can provide valuable, real-world insight into their perceptions of substance use immediately before and during pregnancy.

**Objective:**

The aim of this study was to characterize the frequency and content of posts regarding substance use in the year before pregnancy and during pregnancy among young mothers.

**Methods:**

Facebook posts were mined from young pregnant women (age, 16-24 years) who consented from 2 Midwest primary care clinics that serve a predominantly low-income community. Natural language processing was used to identify posts related to substance use by keyword searching (eg, drunk, drugs, pot, and meth). Using mixed-methods techniques, 2 investigators iteratively coded and identified major themes around substance use from these mined Facebook posts. Outcome measures include the frequency of posts and major themes expressed regarding substance use before and during pregnancy.

**Results:**

Women in our sample (N=43) had a mean age of 21 (SD 2.3) years, and the largest subgroup (21/43, 49%) identified as non-Hispanic black; 26% (11/43) identified as non-Hispanic white; 16% (7/43) as Hispanic; and 9% (4/43) as non-Hispanic mixed race, Native American, or other. The largest subgroup (20/43, 47%) graduated high school without further education, while 30% (13/43) completed only some high school and 23% (10/43) completed at least some postsecondary education. Young women discussed substance use on social media before and during pregnancy, although compared with the year before pregnancy, the average frequency of substance-related posts during pregnancy decreased. Themes identified included craving alcohol or marijuana, social use of alcohol or marijuana, reasons for abstaining from substance use, and intoxication.

**Conclusions:**

Facebook posts reveal that young pregnant women discuss the use of substances, predominantly alcohol and marijuana. Future work can explore clinical opportunities to prevent and treat substance use before and during pregnancy among young, at-risk mothers.

## Introduction

Substance use among young pregnant women is common and a significant public health concern. While pregnancy is associated with significant reductions in alcohol, cigarettes, and other drug use, both alcohol and illicit drug use remain frequent problems during pregnancy. National surveys reveal that among women aged 15-44 years who are early in their pregnancy, 16.5% report alcohol use in the past month, 10.8% report heavy episodic drinking (ie, binge drinking; ≥4 drinks in a row), and 11.5% report illicit drug use. Compared with young adult and adult women, adolescent women report the highest rates of illicit drug use during pregnancy [1].

The American College of Obstetricians and Gynecologists has recommended routine screening and brief interventions for substance use during pregnancy. Marijuana use is emerging as an area of particular concern for childbearing women—for whom marijuana is the most commonly reported illicit drug [1]—given the associated risks for both mothers and infants. For instance, cannabis users are at increased risk of depression, and children exposed to marijuana prenatally have impaired outcomes across several cognitive domains [2,3]. Among a national sample of adolescents aged 12-17 years, 6.5% reported current marijuana use, and 12.0% reported use in the past year [1]. When compared with nonpregnant adolescent girls, pregnant adolescent girls reported rates of marijuana use that were twice as high as nonpregnant peers (6.45% vs 14%, respectively) [4]. Studies have demonstrated that pregnant and nonpregnant women more commonly perceive regular marijuana use as having no risk to their health [5], which may be attributed to remaining areas of uncertainty in the literature regarding effects of marijuana use on the developing fetus, such as fetal growth [6,7]. This discrepancy between perceived safety and physician-identified risk is one of several obstacles that may exist for disclosure of stigmatized behaviors during pregnancy. Technology can help to overcome such barriers. Social media posts can provide insight into young women’s perceptions of substance use that complements data from traditional qualitative research, providing direct observations of their posted views. With the widespread availability of smartphones and internet access, social media has changed the landscape of information gathering and sharing with respect to substance use among adolescents [8,9]. This study aims to characterize the frequency and content of Facebook posts regarding substance use in the year before pregnancy and during pregnancy among young mothers.

## Methods

Facebook posts were mined from 43 young pregnant women (age 16-24 years) who were recruited as a convenience sample and consented from 2 Midwest primary care clinics that serve a predominantly low-income community. The text-based Facebook posts authored by consented women were extracted using the Facebook application programming interface (API). Posts were extracted at study recruitment (typically in the first trimester) and again later in pregnancy (typically in the second or third trimester). At study recruitment, women also provided demographic information, estimated date of delivery, and the date they recalled discovering they were pregnant. During each data extraction, study participants logged on to their Facebook account to grant access, and access was lost once they signed out. Natural language processing was used to identify posts related to substance use by searching posts by keywords (eg, drunk, drugs, pot, and meth) and their morphological variants. Keywords included common synonyms and brand names for alcohol, tobacco, and illicit drugs and were supplemented by internet searching for colloquial synonyms and slang. Additional words were added from synonyms of derivationally related forms from a lexical database, WordNet [10]. Multimedia Appendix 1 provides a full list of keywords that were searched. These identified posts were separated by time stamp into prepregnancy and pregnancy posts. Facebook posts that occurred during pregnancy were identified by a timestamp occurring after the subject’s last menstrual period (LMP), which was imputed from the estimated date of delivery. Facebook posts that occurred within the year prior to pregnancy were identified by a timestamp occurring <1 year prior to the LMP. Substance-related post frequency was compared before and after subjects discovered they were pregnant, using the paired sample *t* test. Using mixed-methods techniques and an inductive framework, 2 investigators (DO and GTW) coded and identified major themes around substance use. Notably, only English-language posts were coded. Codes were derived iteratively, and a formal codebook was established after consensus or discussion between at least 2 investigators, with a third investigator (TC) resolving any disagreements. Posts were not required to identify self-use by the subject to be coded as substance-related. Outcome measures include the frequency of posts and major themes expressed regarding substance use before and during pregnancy. This study was approved by the Institutional Review Board of the University of Michigan (HUM00104989).

## Results

### Quantitative Results

This study included 43 young women aged 16-24 years. Table 1 presents participants’ characteristics. Facebook posts were last extracted at a median of 33 weeks gestational age. Approximately 2% of posts were in Spanish and not analyzed. Facebook posts revealed that young women are discussing the use of substances, predominantly marijuana, alcohol, and tobacco (cigarettes, hookah), before and during pregnancy (Table 2). Overall, 70% (30/43) of subjects posted about substances during the 1 year prior to pregnancy through the end of pregnancy. Furthermore, 56% (24/43) of subjects posted about substances during their pregnancies.

**Table 1:** Demographic characteristics of participants (N=43).

Demographics		Value
**Race (n=43), n (%)**		
	Non-Hispanic black	21 (49)
	Non-Hispanic white	11 (26)
	Hispanic	7 (16)
	Non-Hispanic mixed race or other	3 (7)
	Native American	1 (2)
Age in years, mean (SD); range		21 (2.3); 16-24
**Age group in years (n=43), n (%)**		
	<18	4 (9)
	Between 18 and 21	19 (44)
	>21	20 (47)
**Educational status (n=43), n (%)**		
	Completed some high school	13 (30)
	High school graduate	20 (47)
	Completed some postsecondary education^a^	10 (23)
Median household annual income in US $ (n=28)^b^, median (range)		3800 (0-40,000)
**Household members (n=42)^c^, n (%)**		
	Subject's children	12 (29)
	Parent	11 (26)
	Significant other (boyfriend, fiancé, husband)	11 (26)
	Roommate	5 (12)
	Lives alone	2 (5)
**Relationship status (n=37)^b^, n (%)**		
	Single, never married	16 (43)
	In a relationship but not married	20 (54)
	Married	1 (2)

^a^Subjects younger than 18 years were all counted as completing some high school; 2 of 4 were currently enrolled in school.

^b^Question not asked to participants aged <18 years.

^c^Categories not mutually exclusive.

**Table 2:** The number of substance-related and total Facebook posts.

Substance	Year prior to pregnancy	Pregnancy	Total
Marijuana	82	30	112
Alcohol	78	44	122
Tobacco	11	8	19
Other^a^	6	13	19
Total substance references^b^	177	95	272
Total Facebook posts	9816	13,043	22,859

^a^Heroin, prescription drugs, cocaine, lysergic acid diethylamide, ecstasy, unspecified recreational substances.

^b^Posts sometimes referenced >1 type of substance use.

**Figure 1:**
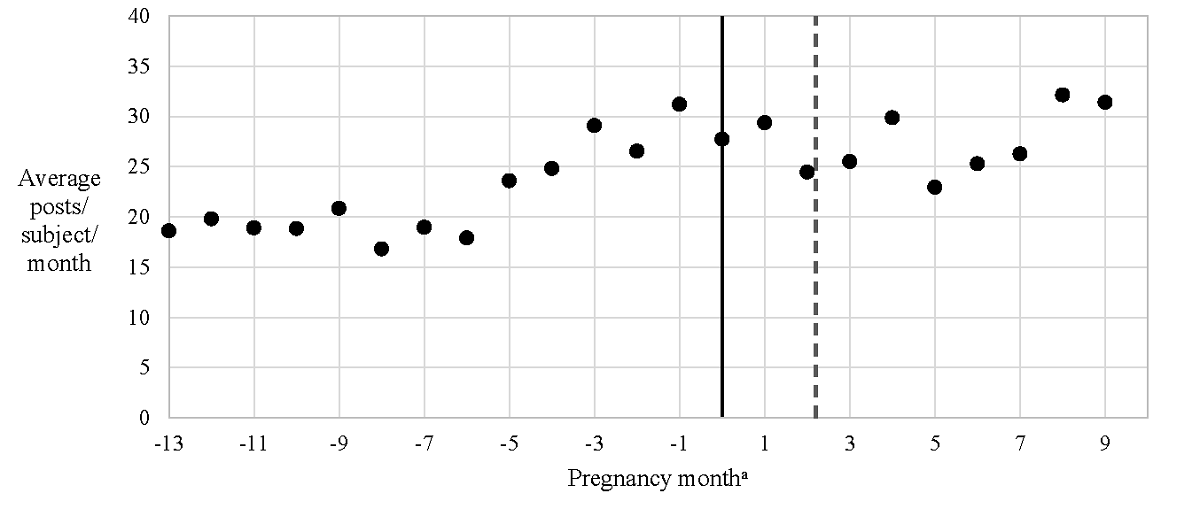
Facebook post frequency in the year before pregnancy and during pregnancy. aPregnancy month is the number of 4-week intervals before or after the last menstrual period. Dashed line, the average time in pregnancy at which subjects reported discovering their pregnancies (each subject reported the date when she discovered she was pregnant during the study intake process; mean 2.2 pregnancy months).

**Figure 2:**
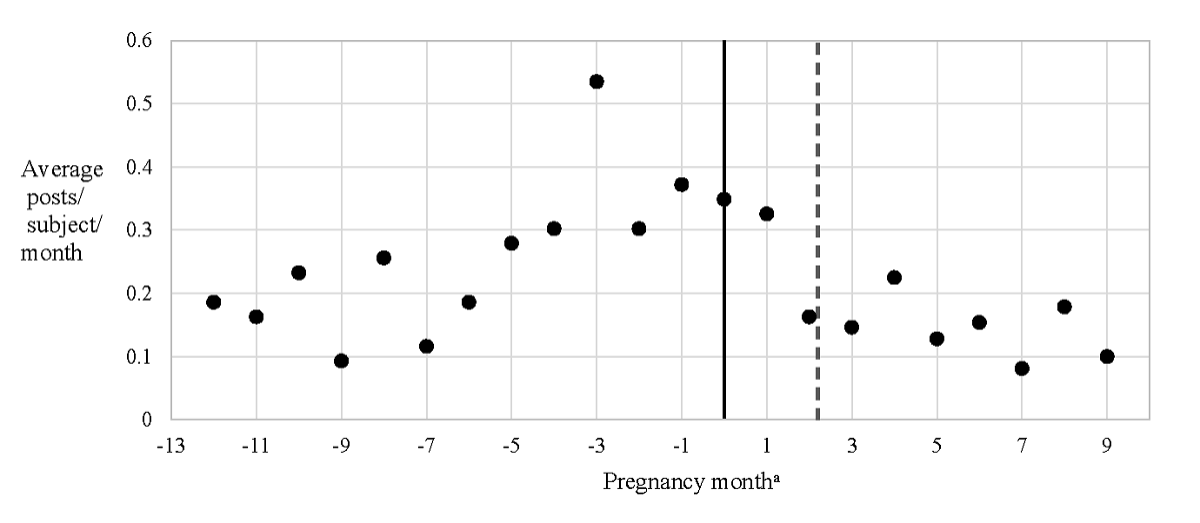
Substance-related Facebook post frequency in the year before pregnancy and during pregnancy. aPregnancy month is the number of 4-week intervals before or after the last menstrual period. Dashed line, the average time in pregnancy at which subjects reported discovering their pregnancies (each subject reported the date when she discovered she was pregnant during the study intake process; mean 2.2 pregnancy months).

The average total Facebook posting frequency (not restricted to posts about substance use) did not appear to change appreciably throughout pregnancy. However, the number of posts about substance use appeared to decrease after pregnancy compared with before pregnancy, most markedly after the date on which participants found out that they were pregnant (Figures 1 and 2). The paired sample *t* test supported a mean difference in substance-related post frequency between the periods before and after subjects discovered their pregnancies (t_42_=2.3, *P*=.03).

### Qualitative Results

Posts that reference substance use in the year prior to pregnancy (Table 3) predominantly focused on use or anticipated use of substances by subjects (118/158, 75% of substance-related posts; “i'm bout 3 shots from drunk tf”). Posts were unlikely to contain an overt value judgment about substance use, but generally referred to substance use in a positive or neutral tone (“I do love my vodka lol”). The most common themes of posts were cravings for substance use (“man i'm up, need to find weed and smoke asap. i fucking hate credit cards bruh”) and references about intoxication (eg, high, drunk, etc; “ima lil tipsy bih right now [4 smiling face with horns emojis]”). Posts describing social substance use (such as use with a friend or at a party) again typically described self-use by subjects (29/32 posts) but often suggested comradery-building (“someone come threw and smoke…i mean shit, we'll become friends! lmao!”).

**Table 3:** Post categories and representative posts in the year before pregnancy. Emojis are denoted by brackets. Beginning of pregnancy defined as the last menstrual period as estimated by the due date. “In a relationship” designates subjects who self-describe as “In a relationship, not married.”

Post categories and subjects		Weeks before pregnancy	Posts
**Cravings for substance use (n=30, average 20 weeks prior to LMP^a^)**			
	Non-Hispanic mixed-race 22-year-old high school graduate, in a relationship	8 weeks	"I swear a blunt would be so legit [face with tears of joy] [face with rolling eyes]"
	Black 19-year-old high school graduate, in a relationship	9 weeks	"I need some weed."
	Black 22-year-old, completed some college, single	10 weeks	"i just want to smoke a fat ass blunt eat some subway and watch movies all day. put in apps on my phone"
	Black 24-year-old high school graduate, relationship status unknown	37 weeks	"I need a drink like right now"
	Black 24-year-old high school graduate, in a relationship	47 weeks	"I feel like drinking a whole bottle of liquor right now [tropical drink]"
**Intoxication (n=32, average 20 weeks prior to LMP)**			
	Black 22-year-old, completed some college, single	15 weeks	"last night I was drunk! af #lilcuzbigcuz [woman’s name]"
	Non-Hispanic mixed-race 22-year-old high school graduate, in a relationship	18 weeks	"I'm so high I ain't never coming down..."
	Black 24-year-old high school graduate, relationship status unknown	24 weeks	"drunk moments with [woman’s name] [face with stuck out tongue and winking eye] [face with stuck out-tongue and tightly closed eyes] [face with tears of joy] [cocktail glass] [tropical drink] [wine glass] [beer mug] [clinking beer mugs]"
	Non-Hispanic mixed-race 19-year-old high school graduate, in a relationship	30 weeks	"I wish I could just drink the pain away but that never works I tend to think about everything when I'm drunk and cry about it"
	Black 16-year-old, enrolled in high school, relationship status unknown	52 weeks	"i'm high ass hell && hungry ass hell im ready to go home"
**Social substance use (n=32, average 16 weeks prior to LMP)**			
	Non-Hispanic mixed-race 22-year-old high school graduate, in a relationship	8 weeks	"sipping on this gen and oj with mines [red heart] [smiling face with heart-shaped eyes] [couple with heart] [smiling cat face with heart-shaped eyes] think i'm a lil tipsy, [man’s name] baby sitting his drink [person’s name] what about you and bro? lol"
	Native American 21-year-old, completed some college, single	10 weeks	"off three hours of sleep and a half pint of henny! but its money to be made, thanks to [man’s name] for turning up at 1am.. i'm so slow motion"
	Black 24-year-old high school graduate, in a relationship	18 weeks	"were my real bitches at like fr i wanna turn up shit if you come over we can have drinks smoke and i'll feed you lol"
	Black 19-year-old high school graduate, in a relationship	14 weeks	"a friendship go two ways just like a relationship if you ain't putting no effort to talk, hang, get high lol whatever the case may be then neither am i fuck it [open hands sign] [100 points symbol]"
	Black 23-year-old, completed some vocational training, single	52 weeks	"i love my cousins...we drunk as fuck!!"
**Nonsocial substance use (n=17, average 25 weeks prior to LMP)**			
	Hispanic mixed-race 20-year-old, completed some high school, in a relationship	8 weeks	"gonna drink this hen dog and watch elf"
	Black 22-year-old, completed some college, single	10 weeks	"why am I drunk by myself? lol"
	Black 19-year-old high school graduate, in a relationship	12 weeks	"just getting off. about to run some bath water, roll up a blunt, & chill [smirking face]"
	Black 24-year-old high school graduate, in a relationship	29 weeks	"it feels amazing to walk into your own home roll a blunt and relax [smiling face with smiling eyes] i hate paying bills but it is worth it"
	Black 20-year-old high school graduate, single	35 weeks	"about to take my daughter to the park then make pepper steak for dinner theeennn sip my wine n a candle lit bubble bath [two women holding hands] [fork and knife] [curry and rice] [wine glass] [bath] [unidentified emoji]"

^a^LMP: last menstrual period.

**Table 4:** Post categories and representative posts during pregnancy.

Post categories and subjects		Gestational age^a^	Did subject know she was pregnant?	Posts
**Negative aspects of or abstinence from substance use (n=30, average gestational age 16 weeks)**				
	Black 22-year-old, completed some college, single	6 weeks	No	"I can't turn up y'all! 4 days no drinking 4 days no squares and 3 days no weed! If you not helpin keep it pushing please and thanks"
	Black 19-year-old high school graduate, in a relationship^b^	14 weeks	No	"y'all don't understand how i miss being turnt up [weary face]^c^ [broken heart] i love my love bug waaaayyyy too much tho! but valentine's day?? it's going tf dowwwnnnn i know my boyfriend gone get me drunk asfffff [weary face] [smiling face with heart-shaped eyes] [smiling face] [face with tears of joy] [face with tears of joy]"
	Non-Hispanic white 18-year-old, completed some college, in a relationship	14 weeks	Yes	"what the fuck????? legalizing heroin? why the hell would anyone legalize heroin. i guess another thing to help population control fucking government."
	Non-Hispanic white 20-year-old high school graduate, in a relationship	16 weeks	Yes	"if you smoke in a car with a pregnant person as your passenger, windows rolled down or not, you're an asshole."
	Black 23-year-old, completed some postsecondary vocational training, in a relationship	31 weeks	Yes	"idk what type of rats yall niggas used 2 but i clearly was raised differently & im not changing who i am 4 nobody. i dont like all types of people in & out my house. especially niggas. i have a daughter. she will never think that shit is ok. if you cant respect that; im not the one 4 you. all that drinking & shit every day/night will not be done in my house. i wasnt raised seeing that shit & my kids wont be neither. yes i smoke weed; if i didnt a lot of mfs wouldnt be breathing so if you dont agree; fck you."
**Social substance use (n=16, average gestational age 14 weeks)**				
	Black 24-year-old, completed some college, in a relationship	3 weeks	No	"seriously tho. we cant hang if i'm the only one high. it doesn't work like that no more unless we're close and you have a good reason for not smoking. lol"
	Non-Hispanic white 22-year-old high school graduate, married	11 weeks	Yes	".. wish i had friends to talk to but i guess that now that i cant drink no one wants to hmu anymore anyways... its kool yall weren't real friends anyway.."
	Non-Hispanic white 19-year-old, completed some high school, in a relationship	26 weeks	Yes	"is anyone interested in coming to a diaper party anywhere from now to 8? bring a pack of diapers and get all you can drink keg and food!!!! in jeremy wayne lambert"
	Black 21-year-old, high school graduate, single	29 weeks	Yes	"lmbo [woman’s name] said im blessed because i told her when [man’s name] take me out to eat im allowed to get a drink and dessert lmbo"
**Intoxication (n=15, average gestational age 22 weeks)**				
	Non-Hispanic white 17-year-old, not enrolled in school, relationship status unknown	6 weeks	Yes	"if you've ever seen me drunk, press like. if i get more than 30, i clearly need help...[face with tears of joy] [clinking beer mugs] haha.."
	Black 23-year-old, completed some vocational training, in a relationship	18 weeks	Yes	"its sad scrolling down my tl & seeing mothers fcked up every weekend. weekdays too smh. your child is going 2 remember you as a drunk. (if they remember you at all b/c you're always putting them on the next while you turn up) but watch these same bitches dog their baby daddies tomorrow on fathers day! smh."
	Non-Hispanic white 18-year-old, completed some college, in a relationship	32 weeks	Yes	"happy 21st birthday to my love [man’s name]. i hope you have a wonderful birthday and dont end up with alcohol poisoning from drinking so much. i love you and i wish you have the best day possible. have a good day![4 face throwing a kiss] [4 party popper]"
	Black 24-year-old, completed some college, in a relationship	34 weeks	Yes	"get high all you want baby girl.!! smoking does kill but we ain't talking about weed.!!"
**Cravings for substance use (n=7, average gestational age 11 weeks)**				
	Black 22-year-old, completed some college, single	6 weeks	No	"who in the green with some weed?"
	Non-Hispanic mixed-race 19-year-old high school graduate, in a relationship	6 weeks	No	"i could go for a shot and a j and a bus bit hey you don't see me doing that so don't tell me shit about #!++!#? a &!@$ that is my!%+@#$"
	Black 23-year-old, completed some vocational training, single	20 weeks	Yes	"i wish i could drink, i'm so irritated!"
	Black 19-year-old high school graduate, in a relationship	21 weeks	Yes	"if i could just smoke a wood.. i wouldnt give a fuck about shit."

^a^Gestational age reported as weeks after the last menstrual period as estimated by the due date.

^b^“In a relationship” designates subjects who self-describe as “In a relationship, not married.”

^c^Emojis denoted by brackets ([ ]).

Fewer substance-related posts during pregnancy (Table 4) described substance use by subjects (28/76, 37%) than in the year prior to pregnancy (118/158, 75%). Of posts describing self-use during pregnancy, the majority was written before subjects discovered they were pregnant (18/28, 64%). The most common theme was discussion of negative aspects of substance use (posts discussing abstinence from substance use were also included in this theme). These posts described the dangers of substance use among pregnant women and their concerns about use around their family or themselves during pregnancy (“i hate it when people who smoke cigarettes come all up in my face and talk to me. if you don't get yo nicotine tobacco smelling ass breath out my face...... ugh.”). Some of these posts demonstrated ambivalence about the types of substance use (“smoking does kill but we ain't talking about weed”) or complete abstinence (“4 days no drinking 4 days no squares and 3 days no weed!”). Another common theme was a discussion of social aspects of substance use. These posts often referred to the social challenges of abstinence (ie, lack of social interaction that includes substance use; “where are the people that know how 2 communicate? can have good conversation w/a sense of humor w/o being drunk or high? too many negative nancy's around here, positive vibes only!”). Furthermore, major themes included craving substances (less common than in the year prior to pregnancy) and references to intoxication (most commonly referring to intoxication in others).

## Discussion

### Principal Findings

We characterized Facebook posts regarding substance use in the year before pregnancy and during pregnancy among a sample of young mothers. We found that the frequency of posts related to substances decreased after subjects discovered they were pregnant; this may represent a decline in the presence of substance use among the lives of young women when they become pregnant or may represent a social stigma of discussing substance use while pregnant. We are not aware of prior research that captures the total or substance-related social media posting frequency throughout pregnancy, though a cross-sectional study showed pregnant women frequently check social media [11].

Pregnancy is a window of opportunity that prompts the majority of women to either reduce or abstain from alcohol and substance use for the remainder of their pregnancy [12]. Many of the substance-related Facebook posts we identified during pregnancy reflected this increased focus on abstinence from and negative consequences of substance use. However, a subset of women continue using substances during pregnancy [1,4]. Our sample of pregnant young women often discussed substance use in ways that highlighted the need for continued interventions to support their abstinence from substances during pregnancy. For instance, women in our sample often expressed a loss of social support because they were not able to participate in the social use of substances; this is similar to a prior qualitative study of Australian women who identified social alcohol consumption as motivation for continued use throughout pregnancy [13]. Unique to this study, some of the posts suggested social abandonment instead of simple loss of social activities: “bitches don't hit you up to check on you if you ain't got a bottle or a blunt.” This finding may reflect a study population with fewer baseline social resources and is amplified by the vivid verbiage found in Facebook posts throughout our sample. Furthermore, although the women in our sample appeared to have knowledge about the need to abstain from substance use during pregnancy, they often expressed some ambivalence about the need to avoid all substances at all times, particularly marijuana. Reportedly, marijuana use during pregnancy is on the rise [14], increasing by 62% over the past decade; this increase has been attributed, in part, to an increased perception of the safety of marijuana use during pregnancy [4,5]. Clinicians should acknowledge the role of substance use in the lives of youth and find ways to ensure these young women are supported and empowered to make healthy decisions during pregnancy.

Disclosure of sensitive topics, such as substance use during pregnancy, is challenging because of social stigma, but can be facilitated by the use of technology. Social media posts by young women can provide valuable insight into their perceptions of substance use during the vulnerable time of pregnancy, with observations unencumbered by a formal research setting. Such information can be used toward preventive efforts in reducing use during pregnancy by identifying the circumstances around why substances are used (ie, social support and addiction) and assist in targeting resources and programs for at-risk mothers.

### Limitations

Although this study represents a novel investigation of substance use, it has a number of limitations. Despite an extensive database of search terms, it is possible that some posts were missed owing to posts with nonsemantic use of words that refer to risk-taking behaviors or novel slang not found during internet searching. However, search terms were identified using a variety of methods that included modern youth-centered vernacular. In addition, some posts related to substance use may be posted through pictures only, which were not analyzed in this study. Posts were extracted at 2 times in pregnancy (at enrollment and a later date in the second or third trimester), and anticipated extraction of posts could have introduced a potential desirability bias; this is felt to be less likely as the research team is only a small part of a much larger audience that would be anticipated to read the posts. Moreover, the quantitative analysis of post frequency is limited by the small sample size and a relatively low proportion of substance-related posts. However, the qualitative data collected provides important nuance and context to these dangerous behaviors among a high proportion of young mothers in our diverse sample. Finally, although our sample included a diverse range of race or ethnicities, findings from our small study may not be generalizable to larger populations of youth.

### Conclusions

Our evaluation of Facebook posts reveals that young pregnant women are discussing the use of substances, predominantly alcohol and marijuana. Providers that care for young pregnant mothers can anticipate and acknowledge the possible loss of social interaction related to substance use and support women in remaining abstinent throughout pregnancy. Future work that explores youth-centered interventions to prevent and treat substance use before and during pregnancy among young, at-risk mothers could improve outcomes for both mothers and their children.

## Appendix

Multimedia Appendix 1 Search terms used to query database of Facebook posts.

## Abbreviations

LMP: last menstrual period
