# Supplementary material for: Substance Use Among Young Mothers: An Analysis of Facebook Posts
Source: JMIR Pediatr Parent. 2018 Dec 4;1(2):e10261. doi: 10.2196/10261 (PMC6716430; doi:10.2196/10261)
Supplement: Multimedia Appendix 1 [file pediatrics_v1i2e10261_app1.pdf]

Appendix: Search terms used to query database of Facebook posts

A. Alcohol-related terms

4 pines brewing company
ab inbev brands
abbotsford invalid stout
absinthe
absolut
adrianna vineyard
advocaat
aglianico
aguardiente
akvavit
albarino
alcohol
ale
alexander keith's brewery
alight
aligote
all or nothing brewhouse
alone
alpestre
altvater
amarcord brewery
amaretto
amarula
amber beverage
amber beverages
amberbock
amstel brewery
anchor
anheuser-busch
anheuser-busch inbev
anisetta
anisette
annedd'ale
ansells brewery
apatin brewery
arak
armagnac
armand de brignac
armaro
arneis
arnott's Shapes
aromatized wine
arquebuse
arrack
artois bock
asgaard das gottliche
assyrtiko
astika beer
atholl brose
atrophied
augustiner brau
aureole
australian max
aventinus
awamori
bacardi
bacardi
baczewski
baderbrau
baijiu
bailey's
baileys
bajtra
ballarat bitter
bananarita
bang up
bankrupt
barbera
barenfang
barenjager
barleywine
baron de lestac
bass brewery
bavarian brewing company
beamish and crawford
beaten track brewery
becherovka
beck's lager
beechleaf
beer
beers
beirao
belhaven best
belle-vue brewery
benedictine
berliner weisse
besotted
betty stogs
beverage
beverwyck brewery
bevo
big rock brewery
big sky brewing company
billy beer
bintang beer
birra ichnusa
birra menabrea
birra menaresta
birra moretti
birrificio angelo poretti
birrificio lambrate
bitburger brewery
black forest devil
black warrior brewing company
blast
blastoff
blaufrankisch
blue moon beer
blue moon beer

blue nun
bluetongue
brewery
boag's brewery
bobal
bock
boddingtons bitter
bodega catena
zapata
boilo
bondi blonde
bonnsch
bony
bootleg brewery
booze
borovicka
borsod brewery
boschendal
boundary bay
brewing company
brahma beer
brandy
brandymel
brasserie saint
james
brava
brew
breznak
brisbane bitter
broo brewery
browar jedrzejow
browar koszalin
browar lomza
bruadar
bud
bud dry
bud extra
bud ice
bud ice light
bud light
bud light lime
bud silver
budweiser
budweiser 66
budweiser
american ale
budweiser
brewmaster's
private reserve
budweiser budvar
brewery
budweiser select
bulmers
bumbo
burgasko
burleigh brewing
company
busch beer
busch ice
bushwacker
buzz
buzzed
by the horns
brewing co.
cab
cabernet
cabernet franc
cabernet
sauvignon
cachaca
caffrey's irish ale
caipivodka
calisaya
camerons brewery
campari
captain morgan
caribou lou
carignan
carling black label
carling brewery
carlton & united
breweries
carlton black
carlton cold
carlton draught
carlton
midstrength
carmenere
carolans
cascade brewery
cascade premium
lager
castel group
castle eden
brewery
castlemaine
brewery
castlemaine
perkins
castlemaine xxxx
cauim
cazalla
centerba
champagne
changeable
chardonnay
charles shaw wine
chartreuse
chenin blanc
chicha
chiswick bitter
chopper heavy
christian moerlein
brewing co.
christian schmidt
brewing company
chug
cider
cieszyn brewery
cinsaut
clix malt liquor
clner hofbrau fruh
cobra beer
cocktail
cocktail mix
cognac
cointreau
cojito
colonial brewing
company
colorado native
lager
colt 45
concha y toro
consume

coopers brewery
coors
coors light
copper coast
wines
copper dragon
brewery
corona
corona discharge
corvina
cotton gin
courage brewery
creme de menthe
cristal
crown lager
crown pilsner
crunked
crush
cuba libre
curious
cynar
cynful
daiquiri
damassine
damiana
dart
dauquiri
dekuyper
demanovka
desi daru
dessert wine
diamond knot
brewing company
disaronno
dogfish head
brewery
dolcetto
dommelsch
brewery
domuz
dooleys
dortmunder actien
brauerei
dortmunder export
dragon stout
drambuie
drewrys
drink
drink in
drinks
drunk
drunkard
dumante
dundee brewing
company
durif
dutch gold
dwersteg's
eblana
ecco domani
einbecker brewery
elbrewery
elysian brewing
company
emotional state
emu brewery
engelika
erdinger
everglo
fall
fat bastard
fat head's brewery
feral brewing
company
fernet
ferrari spumante
field glass
film
firkin brewery
fish brewing
company
flensburger
brauerei
flirtini
foolproof brewing
company
forge
forst
fortified wine
foster's lager
four loko
four seasons wines
frangelico
franzia
frappato
fremont brewing
fritter
fruit wine
fruko-schulz
fuller's esb
furmint
g. schneider &
sohn
gaffel becker & co
gage roads
brewing company
gamay
gambrinus
game day beer
ganter brewery
garganega
gargle
garnacha
genesee brewing
company
genesee cream ale
genny light
gewurztraminer
giggle juice
gimlet
gin
ginjinha
glass
glayva
glaze
glenfiddich
glenturret
glowtini
goof
governator ale
grafenwalder
grain belt
gran gala
grand marnier
grapero

great basin
brewing company
great wall wine
green vesper
greene king ipa
grenache
grey goose
gruner veltliner
guess
guignolet
guinness
guinness black
lager
guinness draught
guinness foreign
extra stout
h. p. bulmer
hacker pschorr
brewery
hahn brewery
hahn premium
hahn super dry
hale's ales
half acre beer
company
hammer
hammered
hard stuff
hare vine
harp lager
harvestmite
hasserder
heart
heineken
heineken
heineken oud
bruin
heineken premium
light
heineken tarwebok
heinrich reissdorf
hennchata
hennessy
henninger brewery
herbsaint
herrenhauser
brewery
hertog jan
hesperidina
himbeergeist
hofmeister lager
holgate brewhouse
holsten brewery
holsten pils
hooch
huangjiu
hudepohl brewing
company
hum
hurricane high
gravity lager
ice harbor brewing
company
ice wine
ignite
illuminated
inbev
inject
injection
intent
intoxicated
iron city brewing
company
iron horse brewery
j. p. chenet
jabuticaba
jack
jack daniel's
jaen
jag
jagermeister
jagertee
james boag's
premium
james ready (beer)
janssen and bechly
brewery
jelen pivo
jeremiah weed
jever
jigger
jiggermast
john smith's
brewery
jose cuervo
joseph schlitz
brewing company
juice
jumbie
jupiler
kahana royale
kahlua
kajmir
kalgoorlie brewing
and ice company
kalimotxo
kamenitza
kannu
kaoliang
karlovacko
kasiri
kb lager
keg
kerrygold
keystone
keystone beer
kilju
kilkenny
killepitsch
killian's
king cobra malt
liquor
king snedley's
beer
kingfisher
kirsch
klosterbrauerei
andechs
knig brewery
knig ludwig
schlossbrauerei
knigsbacher
kokanee beer
kool aid
koskenkorva

krombacher
brauerei
krupnik
kruskovac
kstritzer
kulmbacher
brewery
kummel
kwai feh
labatt 50
labatt brewing
company
lager
lagrein
lagunitas brewing
company
lakeport brewing
company
land shark lager
laser liquor
lasko brewery
lay waste to
lech browary
wielkopolski
leffe
leite de on
leopard's leap
lezajsk brewery
licher
privatbrauerei
lichido
licor
light
light up
lighted
limoncello
lion brewing and
malting company
liqueur
liqueurs
liquor
lit
literature
little creatures
brewery
little world
beverages
liveliness
lobethal bierhaus
london pride
lone star brewing
company
looking glass
lord chesterfield
ale
lowenbrau
lucky lager
lwenbrau
ly shan
ma la sun
mackeson stout
macua
maerzen
maes pils
magic hat brewing
company
maisel brau
bamberg
mai-tai
malbec
malesan
malibu
malt beer
malt shovel
brewery
manischewitz
manx spirit
maotai
maraschino
margarita
margaritas
marsanne
marston's brewery
marston's pedigree
martini
martinis
mash brewing
company
mastica
mastichato
mataro
mateus
matilda bay
brewing company
mcewan's
mead wine
medivka
medronho
meikueilu
melbourne bitter
mencia
merlot
metaxa
methamphetamine
metropolitan
brewing
metzig
mezcal
michelada
micheladas
michelob
michelob bavarian
wheat
michelob golden
draft
michelob golden
draft light
michelob honey
lager
michelob light
mickey's
mike's
miller
miller lite
milwaukee's best
mimosa
mimosas
miodowka
mojito
mojitos
mokatika
molson canadian
molson canadian
67
molson export
monastrell

moosehead
brewery
moth miller
mountain goat
beer
mourvedre
mousel
mouthwash
mouton cadet
muhlen-klsch
muller thurgau
murphy's brewery
muscat
nail brewing
narragansett
brewing company
national bohemian
natural ice
natural light
nebbiolo
negroamaro
negroni
nero d'avola
neutralize
new albanian
brewing company
new glarus
brewing company
newcastle brown
ale
nihamanchi
nikolaschka
nip
nocello
nocino
north american
breweries
nt draught
odin brewing
company
oettinger beer
off color brewing
ogidiga
ogogoro
okocim brewery
oland export ale
old milwaukee
old speckled hen
old style pilsner
old vienna
olde english 800
orahovac
orange wine
orangerie
oranjeboom
brewery
organic honey dew
oriental brewery
otiose
ouzini
ouzo
ozujsko
paan
pabst blue ribbon
palinka
parakari
party
patron
patzaran
paulaner brewery
peeterman artois
pernod
peroni brewery
peter grant hay
petit verdot
petite sirah
photograph
picaroons
traditional ales
pilsener
pilsner urquell
pimm's cup
pina colada
pina coladas
pine away
pino
pinot blanc
pinot grigio
pinot gris
pinot meunier
pinot noir
pinotage
pint
pipeworks
brewing
pisang ambon
pisco
piscola
piton beer
plum jerkum
poire williams
poison
poitin
ponche
porchcrawler
potty
poulsard
power's brewery
primitivo
privatbrauerei
wittingen
private stock (malt
liquor)
prosecco
pulque
pure blonde
pyramid breweries
queensland
brewery ltd
radeberger
brewery
radegast
rakia
ratafia
red bicyclette
red dog beer
red oak beer
red stripe
red white & blue
beer
red wine
redhook ale
brewery
refreshment
resch's

rheingold beer
ribolla gialla
riesling
riunite
rivulet
rocket republic brewing company
rogue ales
rogue beard beer
rohrbach brewing company
rolling rock
rompope
rose wine
rossbacher
rothaus
roussanne
royal club
rum
rummy
rumpleminze
sagrantino
sagres beer
sahti
sake
sakura
salmiakki
sambuca
samuel adams
sangiovese
sangria
sangsters
sankt laurent
sauce
sauvignon
sauvignon blanc
scene
schaefer beer
schooner lager
schwarzbier
scottish & newcastle
scrumpy jack
sedi daru
semillon
sgc
shan hibiscus
shan lotus
shan rose
shandy
shine
shiraz
shochu
shock top
shoot
shooting
shot
shot glass
sidi brahim
sierra nevada brewing company
silvaner
singani
skinny blonde
slavena beer
slivovitz
small town brewery
smash
smashed
smirnoff
smithwick's
sn tinh
snapshot
snare
soju
solo
sombai
sonti
south australian brewing company
southwark bitter
sparkling wine
spaten-franziskaner-brau
spirit
spirits
squandered
st arnou
st. erhard
st. hubertus
st. ides
st. pauli girl
staatliches hofbrauhaus in munchen
stadtbrauerei spalt
stampede light
starobrno brewery
staropramen brewery
steel reserve
steelback brewery
stella artois
stolichno
stone & wood brewing co.
stones bitter
stout
straw wine
strega
stroke
strongbow cider
surinaamse brouwerij
swallow
swan brewery
sweetwater brewing company
swish
syrah
table wine
tamagozake
tannat
tavernello
taxi
tear
tempranillo
tennent's lager
tennent's super
tepache
tequila
tetley's brewery
theakston brewery
thunder road

[illegible]
tiswin
toast
tonic
tooheys brewery
tooheys extra dry
tooheys new
tooheys old
tooheys pils
tooth and co.
top totty
torrontes
touriga nacional
trapiche
trebbiano
triple sec
truman's brewery
tschunk
tsipouro
tuaca
tuica
tusker
tyskie
ugni blanc
uhudler
ultraviolet
[illegible]

[illegible]
visinata
vitamalz
vodka
vodkas
warka brewery
warsteiner
waste
wasted
webster's brewery
wernesgruner
west coast cooler
west end draught
weyerbacher brewing company
whiskey
whiskeys
whisky
whitbread
white house honey ale
white wine
wild goose beer
williamine
windhoek lager
wine
wisniowka
witbier
woodpecker cider
worthington's white shield
xtabentum
xxxx bitter
xxxx gold
xxxx summer bright lager
yakima brewing
yellow tail
yuengling
zagorka
zedazeni
ziegenbock
zin

[illegible]
zubr
zurawinowka
zurracapote
zweigelt
zywiec brewery

B. Tobacco-related terms

black & mild
camel
chew
cigar
cigarette
cig
dutch masters
e-cigarette
e-cig
kool
marlboro
maverick
newport
pall mall
pyramid
smoke
santa fe
snuff
swisher
vape
white owl
winchester
winston

C. Illicit substance-related terms

420
adderall
addy
antifreeze
articulation
bake
balmy
batch
bath salts
beans
bermuda grass
big h
blaze
blaze away
blazed
blitz
blitzed
blunt
bong
bowl
bowling ball
broil
burn a stick
burn one
cat valium
chalk
chasing the tiger
classy
cockamamie
cola
crank
cranking
crunking
d
deaden
denounce
dex
dextromethorphan
dilaudid
dm
doing up
doob
doobie
dope
drex
dull
dumbbell
e
eatage
ecstasy
eminent
fastball
fattie
fent
fentanyl
fleeceable
fume
gamey
ganja
getting fried
getting glassed
glass tweak
go fast
going on the nod
grass
green
greens
herb
heroin
high
high gear
hopped-up
hotbox
hug drug
hydro
ice
indo
irregular
joint
junk
k
ketamine
kibbles & bits
killers
lean
love drug
marijuana
mary jane
mdma
mephedrone
meth
mj
narcotic
narcs
numb
o.c
opioids
oxycontin
painkillers
park
pharmies
pharms
pit
plant food
pot
potentiometer
psychedelic
heroin
purp
purple drank
reefer
ritalin
roast
robo
robodosing
robotripping
rojo and tussin
roll
roll of tobacco
senior high school
shooting up
ska
skag
smack
smoke
sniff
special k
speed crystal
spinning
stadium
stone
stoned
super acid
supergrass

syrup
thunkder
tobacco
tobaccy
toilet
toke up
tweaking
vic
vicodin
vitamin d
vitamin k
wacky
weed
x
xanax
xanibars
xtc
